# Spherical Confinement of Chromonics: Effects of a Chiral Aminoacid

**DOI:** 10.3390/nano12040619

**Published:** 2022-02-12

**Authors:** Lorenza Spina, Federica Ciuchi, Caterina Maria Tone, Riccardo Barberi, Maria Penelope De Santo

**Affiliations:** 1 Physics Department, University of Calabria, Ponte Bucci, Cubo 31C, 87036 Arcavacata di Rende, Italy; loryspina@libero.it (L.S.); caterina.tone@fis.unical.it (C.M.T.); riccardo.barberi@fis.unical.it (R.B.); 2 CNR-Nanotec c/o Physics Department, University of Calabria, Ponte Bucci, Cubo 31C, 87036 Arcavacata di Rende, Italy

**Keywords:** chromonic liquid crystals, chiral self-assembly, X-ray diffraction, functional materials

## Abstract

Induced or spontaneous chirality in natural systems is an intriguing issue. In recent years, a lot of attention has been focused on chirality of chromonic liquid crystals, a class of materials that is able to self-assemble in columnar structures. However, the mechanism involved in the arising of chirality in these materials, that starts at the molecular level and controls the supramolecular structure, is poorly understood; however, it is certainly affected by ionic strength. In this work we present the results obtained doping Cromolyn, a chromonic material, with a strong helical-twisting-power peptide, and confining it in a spherical geometry. We demonstrate, by means of optical polarized microscopy and structural analysis, that both the geometrical constraint and the presence of the chiral dopant enhance the chiral effect; we also demonstrate that they favor the rise of a highly ordered helical superstructure, that may be optimized upon adding an ionic dye to the system. Finally, we report a procedure for the preparation of free-standing polymeric films, embedding and preserving the microspheres, and paving the way for the creation of biocompatible and eco-friendly optical devices to be used in the sensor and anticounterfeiting fields.

## 1. Introduction

Lyotropic chromonic liquid crystals (LCLCs) are a particular class of liquid crystals in which mesophases appear to vary both with the solvent concentration, usually water, and the temperature. Chromonic molecules have a disk-like polyaromatic central core and two or more ionic groups at the peripheries. Through non-covalent interactions, such as van der Waals, electrostatic, excluded volume, hydrophobic and pi–pi stacking, molecules are able to self-assemble, even at low concentrations, in cylindrical supramolecular aggregates. These, in turn, self-align along a common direction, the director, giving rise to nematic or 2D phases. Food dyes, drugs, and DNA and its derivatives belong to this class of materials. Cromolyn (DSCG), an anti-asthmatic drug, is an example of a LCLC whose phase diagram and properties are well-known.

Chirality is a widely studied phenomenon in thermotropic liquid crystals (TLCs) that can be easily induced by doping a nematic with chiral moieties. Chiral nematic liquid crystals have been investigated in detail when confined in planar and spherical geometries. 

This last form of confinement is obtained by dispersing the liquid crystal in an immiscible fluid and subjecting it to mechanical stirring to obtain an emulsion of liquid crystalline microspheres.

The torque imposed by the chiral molecules on the nematic ones gives rise to a helical arrangement of the director inside the microsphere that provides them with peculiar optical properties. A plethora of stable and metastable optical textures is observed that can also be easily modulated by external stimuli. As an example, when planar anchoring is imposed at the interface and the radius of the microsphere is larger than the pitch of the cholesteric helix, the typical Frank–Pryce texture is obtained. It consists of a series of concentric layers with a defect at the center, with the helical axes oriented radially. If the pitch of the helix is comparable with the wavelength of visible light, Bragg type reflection is observed from microspheres. This optical feature can be exploited in applications in photonics [1], sensing [2] and anti-counterfeiting [3]. 

In this last context, the intricate and difficult-to-reproduce textures obtained in microspheres when subjected to external stimuli [4,5] makes them ideal candidates for the creation of novel anti-counterfeiting tags to be used for tracing, authentication and identification of goods.

With respect to thermotropic liquid crystals, LCLCs offer the considerable advantage of being biocompatible and eco-friendly. LCLCs when confined in curved geometries, for example, in tactoids, droplets, and capillaries, show large reflection symmetry breaking [6]. This is due to the fact that the strong splay and bend deformations of the director, induced by the curved interface and the surface anchoring at this interface, are relaxed through twist deformations [7,8]. This natural chirality may be enhanced by doping them with L and D peptides [9,10]. Tactoids or microspheres are an easy method to evaluate chirality in chromonics with respect to standard fingerprint texture or the Cano-wedge method [6,11]. In general, without chiral additives, the number of tactoids that show left or right asymmetry are equal to each other; conversely, when the chiral material is added, one type of tactoid prevails. The observed cholesteric pitch can vary from two microns to tens of microns depending on the helical twisting power of the peptides and on their concentration. Recently, a pitch of around three microns was obtained in a planar geometry doping DSCG with trans-hydroxyproline (Trans-Hyp) [11]. The possibility to further reduce the pitch is appealing from a fundamental point of view, but especially for applications, to enhance the material’s optical and photonic properties. Chiral textures in microspheres are also easily measurable with a polarized-light optical microscope (POM), since in curved geometries, chirality is straightforwardly detectable, especially for high pitch values [12]. The textures observed in chromonics [13] are similar to the ones reported in several papers focused on chiral TLCs, even if LCLCs contain different ingredients that are thermodynamically balanced in the mesophases with respect to TLCs. Loss of water, temperature variations, changes in dopant solubility and the use of ions can affect the phase diagram and, consequently, the droplet state diagram [5]. For this reason the induction of chirality in LCLCs is extremely difficult to control and a deeper understanding of the mechanisms that control the supramolecular self-assembly at molecular level is mandatory.

The work reported here is focused on the properties of DSCG, confined in microspheres and doped with two different amino acids with different helical twisting power: L-alanine and Trans-Hyp. As for TLCs the spherical confinement is obtained preparing an emulsion of chiral DSCG in a suitable immiscible matrix. 

As discussed previously, the easiest procedure to obtain an emulsion is to confine the water-based chromonic solution in an oily matrix and to shake the blend. In TLCs, microfluidic techniques are used to create an emulsion of monodispersed liquid-crystalline microspheres. For LCLCs this technique is extremely difficult to implement since other materials, as surfactants, have to be added in order to decrease the interfacial tension between the two immiscible liquids, and this can alter the system’s thermodynamic balance [14,15]. Further, the oily matrix must also have low viscosity to be pumped inside microfluidic channels, and this poses a limitation on the materials that can be used. Once the emulsion is prepared, the textures obtained in microspheres, that are the fingerprint of the molecular arrangement, are observed by POM. Experimental analysis confirms the presence of the typical radial arrangement of the Frank–Pryce texture indistinguishable from the one obtained for thermotropic liquid crystals. Thin films of the chiral material are studied as well to obtain information on the chirality induction mechanism; the results confirm what has been previously observed for L-alanine-doped DSCG. In addition, in the case of Trans-Hyp, it appears clear that the mechanism involved in induced chirality is an external binding of the chiral molecules to DSCG cylinders [13]. Nevertheless, in the reported case, the presence of Trans-Hyp considerably increases the number of microspheres that exhibit the ordered texture. Further, the effect of a charged fluorescent dye on the texture formation was also investigated. 

The emulsion is extremely useful since it offers the opportunity to easily manipulate the different components, and provides the system with a high reconfigurability. Nevertheless, a fluid matrix is not optimal for applications because water slowly evaporates from the microspheres. Transforming the mixed emulsion in a polymeric film in which microspheres are embedded is a necessary step to preserve their optical properties in time [16]. Microspheres containing a LCLC were, for the first time, successfully incorporated in a solid matrix in order to be used in practical applications. The texture obtained in the microspheres’ emulsion is preserved in the free-standing polymeric films and, if the system is kept at low temperatures, it is stable for days. When the system is brought to room temperature the texture degrades after a few days. This makes the investigated materials suitable as anti-counterfeiting labels for cold-chain security applications [17,18,19].

## 2. Materials and Methods

### 2.1. Chromonic Liquid Crystal

**Figure 1:**
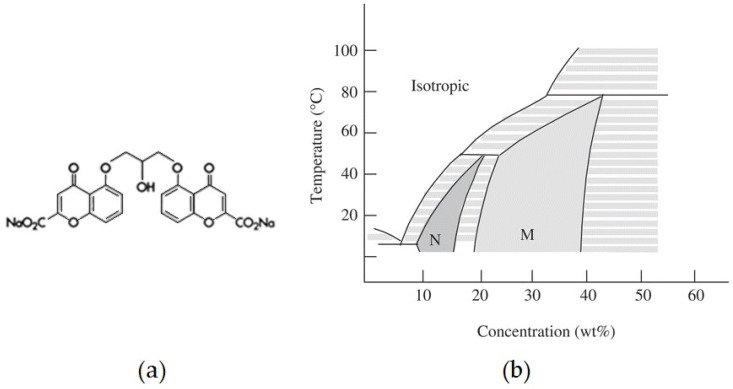
(**a**) Molecular structure of the anti-asthmatic drug, disodium cromoglycate; (**b**) phase diagram of the chromonic DSCG/water system.

DSCG (Figure 1a) was purchased from Sigma-Aldrich (Steinheim, Germany) and used without further purification. When dissolved in water it stacks in cylinder formations, the length of which depends on temperature and concentration; the cylinders then give rise to a nematic phase. At room temperature, the nematic phase exists between 12 and 16 wt% (Figure 1b) [20].

**Figure 2:**
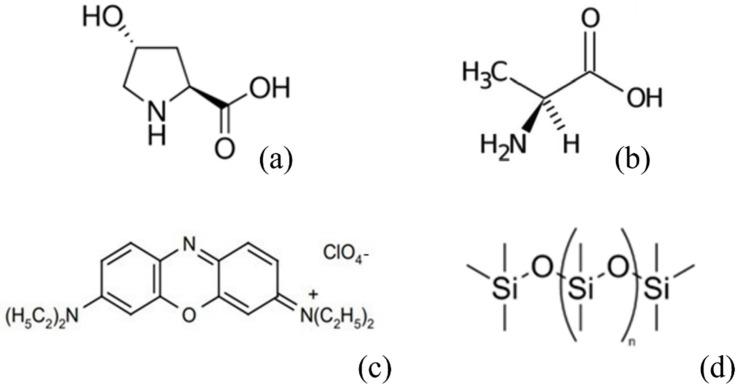
Molecular structure of: (**a**) Trans-4-Hydroxy-L-proline; (**b**) L-alanine; (**c**) oxazine 725; and (**d**) Polydimethylsiloxane.

Trans-4-Hydroxy-L-proline (Trans-Hyp) was purchased from Sigma-Aldrich (Figure 2a), and a solution of 26% of Trans-Hyp in ultrapure water was used to prepare chiral DSCG.

L-alanine, a non-polar left-hand amino acid, was purchased from Sigma-Aldrich (Figure 2b). For the chiral mixture, DSCG was dissolved in 10% *w*/*w* L-alanine water solution. 

Oxazine 725 (Exciton, Lockbourne, OH, USA) is a fluorescent dye, with a positive charge. In water, it has an absorption peak at 650 nm and an emission peak at 725 nm. Its molecular structure is shown in Figure 2c. It was added to chromonic solution in a percentage less than 1‰.

### 2.2. Chromonic-in-Oil Emulsion

To confine the LCLC in a spherical geometry, emulsions were prepared adding a small amount of DSCG to an immiscible matrix. In particular, polydimethylsiloxane (PDMS) was used. PDMS (Sylgard kit 184, Sigma-Aldrich) is liquid, optically clear and has a high viscosity. The kit is composed of the material and the crosslinker. The PDMS structure is shown in Figure 2d. It was used as received. 1ml of PDMS was placed at the bottom of a vial, and a droplet of DSCG was successively added to prevent its adhesion to wall surfaces. Through mechanical agitation, a large number of microspheres were obtained.

The volume fraction of chromonic solution in oil was ~1%, and the resulting droplets had diameters ranging from 1 to 300 μm. For microdroplet texture investigations, 10 μL of the prepared emulsion were sandwiched between two laboratory glass plates separated by mylar stripes. Glasses were then glued using epoxy resin to avoid emulsion leakage and slow down water evaporation. Textures were studied using a polarized light microscope (DMRX, Leica, Wetziar, Germany).

### 2.3. Thin Films 

DSCG and chiral DSCG thin films were prepared as in the following. Initially, glasses were thoroughly washed to remove dust and oily residues; then, they were dried with a jet of hot air. A thin polymeric layer of PDMS with crosslinker was deposited by spin-coating on the glasses and, to eliminate any solvent residue, they were placed in an oven at 130 °C for 30 min. In order to increase the wettability of the polymeric substrate and, therefore, to favor the deposition of a uniform and thin film, the coated glasses were treated for two minutes in a plasma cleaner. Once the substrates were ready, 5 μL of chromonic solution was deposited on each of them. Samples were kept at 4 °C and at controlled humidity for one week; then, they were brought back to room temperature and analyzed.

Thin films were measured by X Ray diffraction (XRD) by a D8 Discover (Bruker Axs, Karlsruhe, Germany), λ = 1.5418 Å using two different geometries (Bragg Brentano and Asymmetric Geometry), and by Atomic Force Microscopy (AFM) using a Multimode 8 (Bruker, Santa Barbara, CA, USA) equipped with a Nanoscope V controller operating in QNM mode. Topographies were acquired using a silicon nitride tip from Bruker, with k = 40 N/m and a radius 10 nm. 

### 2.4. Free-Standing Flexible Films 

Flexible free-standing films, in which microspheres containing nematic and chiral solutions of DSCG were embedded, were obtained using the following procedure. The starting emulsions were prepared as described above with the only exception being in the case of PDMS, wherein the hardener was added in a proportion of 9:1. Emulsions were then deposited in small glass containers and left to dry for 3 days at room temperature. The transparent and flexible films were removed from the containers and observed using POM, they were stored either at room temperature or at 4 °C.

## 3. Results and Discussion

As known from the literature, Trans-Hyp has a greater twisting power than L-alanine (0.75 μm^−1^·wt%^−1^ for L-alanine and 2.5 μm^−1^·wt%^−1^ for Trans-Hyp) [10,11]. Based on previous experience with emulsions of DSCG doped with L-alanine [13] the results presented in this paper indicate that the addition of the Trans-Hyp allows us to obtain a shorter pitch with respect to the one observed in L-alanine-doped samples. A well-defined Frank–Pryce structure is observed. 

### 3.1. POM Measurements

**Figure 3:**
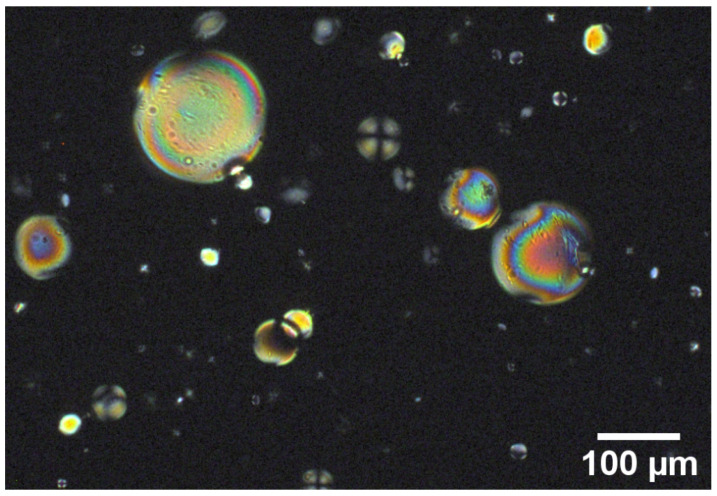
Pure DSCG confined in microspheres and observed by POM.

Microspheres contained in the emulsion have different sizes (ten to hundreds of microns). It is well known from the literature that PDMS provides a homogeneous homeotropic alignment at the interface [21]. When pure DSCG is confined in microspheres, we observe different textures related to their size. In particular, bipolar textures are observed in large droplets, while small ones exhibit the typical Maltese cross (Figure 3).

**Figure 4:**
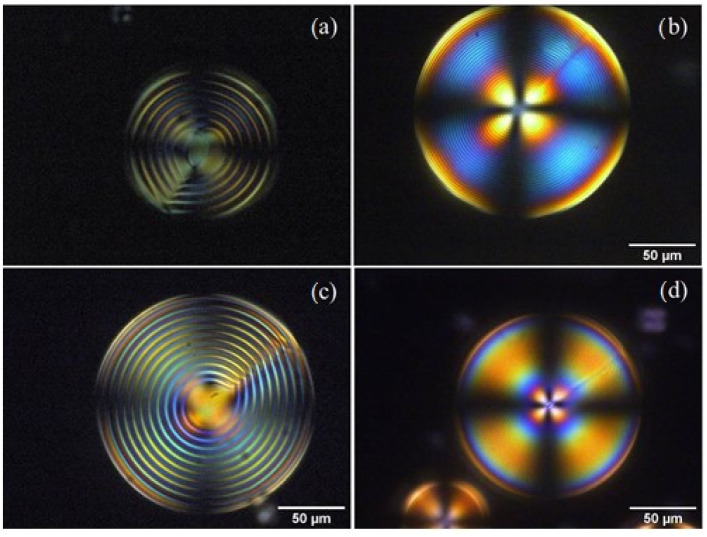
POM image of a microsphere of: (**a**) DSCG + L-alanine; (**b**) DSCG + Trans-Hyp; (**c**) oxazine-doped DSCG + L-alanine; and (**d**) oxazine-doped DSCG + Trans-Hyp.

In chiral doped DSCG, the observed texture is the one typically observed in chiral thermotropic liquid crystals with a radial configuration of the helix axes and a radial disclination line, usually referred to as the Frank–Pryce texture. For comparison, Figure 4a shows a POM image of a sphere containing a mixture of DSCG and L-alanine, and Figure 4b shows an image of a sphere containing a mixture of DSCG and Trans-Hyp. It is well-visible that the pitch has largely decreased due to the high chiral twisting power of Trans-Hyp compared to that of L-alanine. What is also interesting to underline is that the birefringent core is not observed in the microsphere containing Trans-Hyp, whose imaged area is completely covered with concentric rings. In this case, the chromonic Frank–Pryce texture is indistinguishable from the one observed in chiral nematic thermotropic liquid crystal.

With respect to emulsions prepared doping DSCG with L-alanine, the ones prepared using Trans-Hyp show a larger number of microspheres with well-defined Frank–Pryce textures.

**Figure 5:**
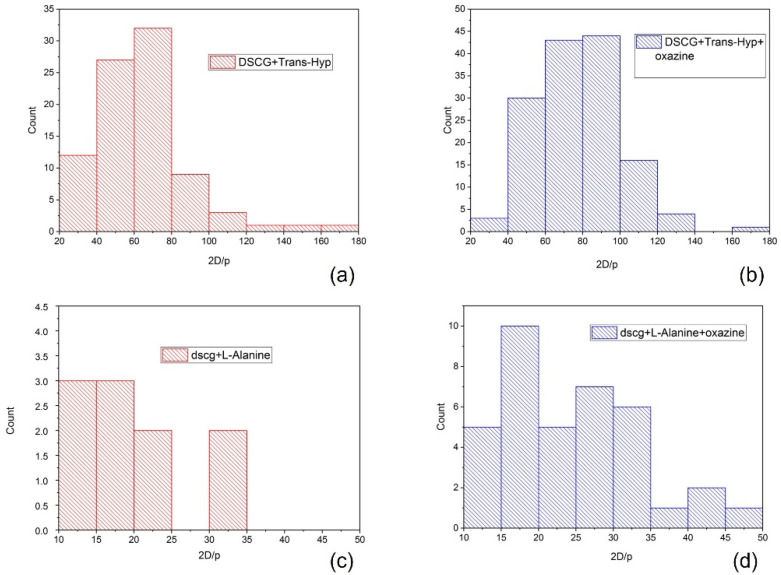
Chirality parameter distribution for: (**a**) DSCG + Trans-Hyp; (**b**) oxazine-doped DSCG + Trans-Hyp; (**c**) DSCG + L-alanine; and (**d**) oxazine-doped DSCG + L-alanine.

The average half-pitch measured is 1.65 ± 0.10 μm, in agreement with reference [11], which is considerably shorter with respect to the one measured on microspheres doped with L-alanine of 5.20 ± 0.20 μm. Figure 5a highlights the microsphere distribution considering the chirality parameter 2D/p [19,22]. This parameter is largely used in chiral thermotropic liquid crystals to classify the textures observed in microspheres and, in the present case, it can help in highlighting pitch variations as a function of the sphere geometry [5]. 

From previous observations [13] we have found that oxazine 725 improves the quality of the Frank–Price texture (Figure 4c and 4d). Figure 5c and Figure 5d show, in the case of DSCG containing L-alanine, that the number of microspheres with a well-defined texture increases when the material is doped with oxazine. Using the same approach, we doped DSCG containing Trans-Hyp with oxazine725 (Figure 5b).

In this case, a slightly lower average half-pitch was measured, 1.50 ± 0.10 μm, with respect to what observed for DSCG doped with Trans-Hyp alone (Figure 5a). This is clearly visible from the histogram shown in Figure 5b, which is shifted to higher values of the chirality parameter. In addition, the number of analyzed spheres is considerably larger for the oxazine-doped sample with respect to the non-doped one, which confirms the fact that the small ionic dye molecules help in inducing the ordered texture. The histograms related to the L-alanine samples show the same behaviour.

Unfortunately, the Frank–Pryce texture, if microspheres are stored at room temperature, deteriorates after approximately 2 weeks, the degradation time depending on the temperature and humidity of the environment. This process is not reversible. If refrigerated at 4 °C, the texture is preserved for two weeks.

### 3.2. AFM Measurements

**Figure 6:**
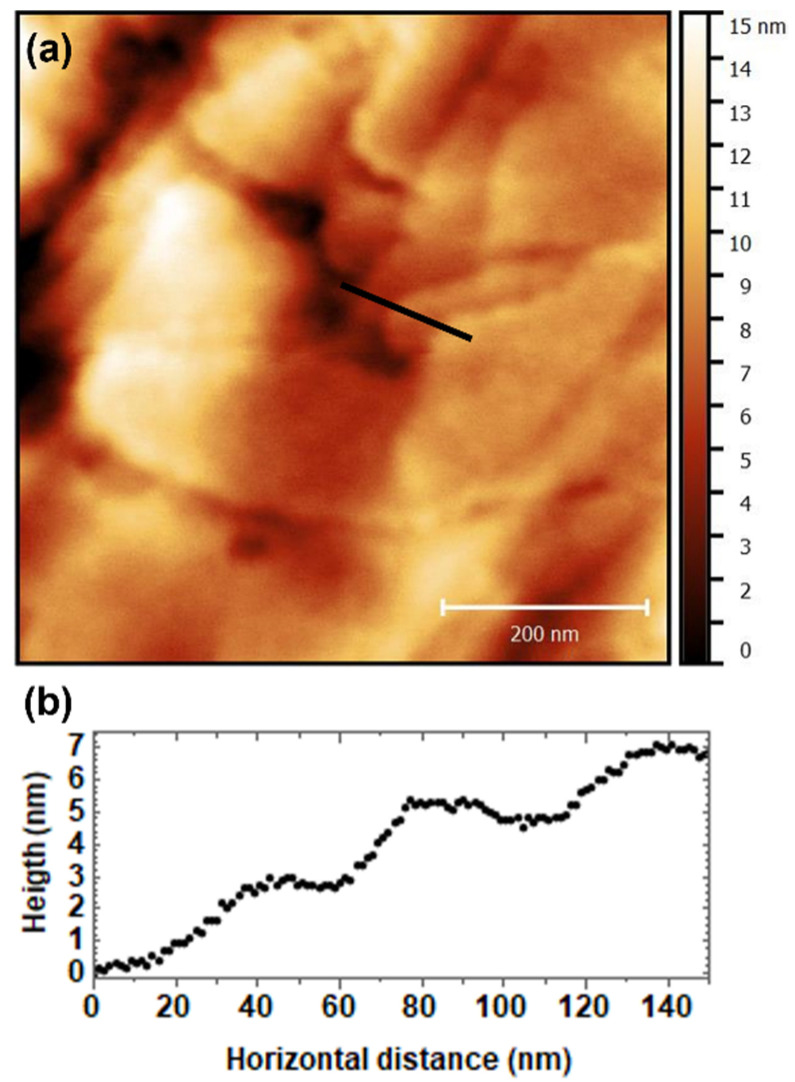
(**a**) AFM topography of DSCG doped with Trans-Hyp; and (**b**) line profile of the terraces on the surface.

To gain information on the molecular packing, thin films of dried DSCG doped with Trans-Hyp and deposited on glass were analyzed through Atomic Force Microscopy. The surface appears to be homogeneous on a large scale (3 × 3 µm^2^) with a roughness of about 20 ± 4 nm. When imaged on smaller scales (Figure 6a), the surface shows the presence of terraces with a height of 2.7 ± 0.4 nm (Figure 6b), and the mean roughness decreases to 2.8 ± 1.1 nm.

As reported in reference [13], DSCG is able to organize in a layered structure; this tendency is confirmed when it is doped with Trans-Hyp, indicating that this compound does not affect the chromonic self-assembly. 

### 3.3. X-ray Measurements

**Figure 7:**
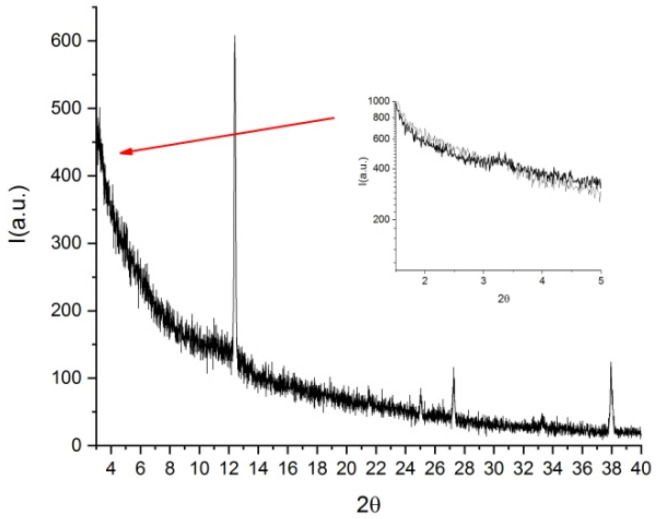
X-ray diffractogram of DSCG doped with Trans-Hyp.

The analysis of thin films by POM shows crystal formations in the sample. While at XRD low angles, a peak corresponding to 27 Å is hardly visible (Figure 7), at high angles, narrow peaks related to Trans-Hyp are clearly visible; this confirms the phase separation and suggests that the chiral dopant is not intercalated in DSCG columns [23]. The DSCG peak at the high angle is scarcely visible. The 2D order of DSCG is partially lost during the film drying differently from the pure DSCG, which maintained a hexagonal 2D phase [13]. 

### 3.4. Free-Standing Film

**Figure 8:**
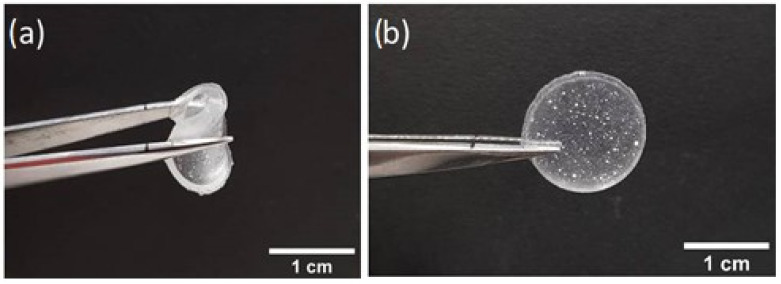
Flexible film (**a**) folded and (**b**) unfolded.

Microspheres were successfully trapped in a polymeric free-standing film. Emulsions were prepared using DSCG doped with both L-alanine and Trans-Hyp, and poured in small circular vessels about 1.5cm in diameter. The thick layers were then left to dry at room temperature. Figure 8 shows the film obtained after the above-described procedure.

**Figure 9:**
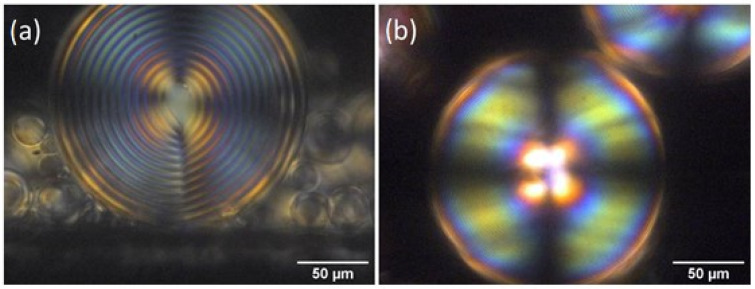
Polarized-light optical microscopy image of a microsphere of: (**a**) oxazine-doped DSCG + L-alanine; and (**b**) oxazine-doped DSCG + Trans-Hyp.

Due to the rubbery matrix, microspheres in both cases appeared opaque (Figure 9). 

**Figure 10:**
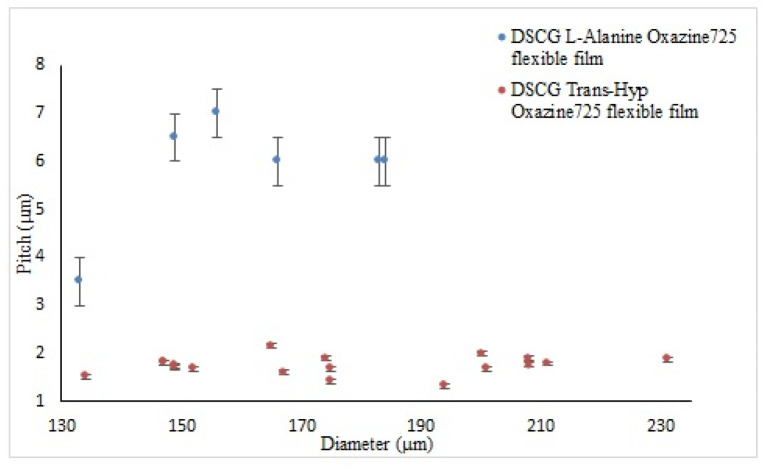
Cholesteric half-pitch as a function of microsphere diameter for oxazine-doped DSCG + L-alanine (blue), and oxazine-doped DSCG + Trans-Hyp (orange).

Comparing data from the two flexible films (Figure 10), the half-pitch observed in microspheres containing DSCG doped with L-alanine had an average value of 5.83 ± 0.05 μm, whereas in the sample with Trans-Hyp, a half-pitch of 1.74 ± 0.05 μm was measured. These values are comparable with the ones measured in emulsions. 

Storage at 4 °C allows maintenance the textures for at least 10 days, after which the microsphere degradation process begins.

## 4. Conclusions

Being able to obtain well-defined optical textures is the first crucial step towards the use of chromonic liquid crystals confined in microspheres for practical applications. In this work, for the first time, we have shown that a Frank–Pryce texture, indistinguishable from the one observed in TLC microspheres, can be obtained by doping a chromonic with an high-twisting-power amino acid. What happens at the molecular level seems to be consistent with what is observed for amino acids with low helical-twisting power. The chiral dopant does not influence the molecular stacking of DSCG, but it attaches to the external part of the cylinders, helping their twist, which is also favored by the spherical confinement.

The half-pitch obtained using Trans-Hyp is around 1.65 microns. This value is still too big for applications that exploit the use of visible light in devices. Nevertheless, it represents an important step toward this direction and applications in the sensor field could already be within reach.

The addition of an ionic dye to the solution improves the quality of the texture, and the pitch measured is slightly lower than the one obtained using Trans-Hyp alone. As reported in a previous article [13], a similar effect has been observed by adding a 1M solution of a monovalent salt to the chromonic mixture. It is known that the presence of ions causes an elongation of the columnar aggregates due to electrostatic effects. In this case, since the dye quantity is in the mM range, we may ascribe the effect both to electrostatic and steric interactions.

The preservation of the microsphere texture when embedded in a thin flexible film has never been reported before. This was obtained by preparing the emulsion with PDMS mixed with its hardener. The possibility of preparing biocompatible and eco-friendly flexible films is fundamental for the creation of novel devices. Due to the temperature sensitivity of the texture, at present, its applications in food cold-chain tracing and sensor technology are envisaged.

## Data Availability

The data presented in this study are available on request from the corresponding authors.

## References

[1] Muševič I. (2016). Liquid-crystal micro-photonics. Liq. Cryst. Rev..

[2] Petriashvili G., Bruno M.D.L., De Santo M.P., Fuoco E., Barberi R. (2018). Acid mediated tunability of stimulated laser emission from dye doped chiral microdroplets. Mol. Cryst. Liq. Cryst..

[3] Geng Y., Noh J., Drevensek-Olenik I., Rupp R., Lenzini G., Lagerwall J.P.F. (2016). High-fidelity spherical cholesteric liquid crystal Bragg reflectors generating unclonable patterns for secure authentication. Sci. Rep..

[4] Orlova T., Aßhoff S.J., Yamaguchi T., Katsonis N., Brasselet E. (2015). Creation and manipulation of topological states in chiral nematic microspheres. Nat. Commun..

[5] Krakhalev M.N., Rudyak V.Y., Prishchepa O.O., Gardymova A., Emelyanenko A.V., Liu J.-H., Zyryanov V.Y. (2019). Orientational structures in cholesteric droplets with homeotropic surface anchoring. Soft Matter.

[6] Peng C., Lavrentovich O.D. (2015). Chirality amplification and detection by tactoids of lyotropic chromonic liquid crystals. Soft Matter.

[7] Zhou S., Neupane K., Nastishin Y.A., Baldwin A.R., Shiyanovskii S.V., Lavrentovich O.D., Sprunt S. (2014). Elasticity, viscosity, and orientational fluctuations of a lyotropic chromonic nematic liquid crystal disodium cromoglycate. Soft Matter.

[8] Jeong J., Davidson Z., Collings P.J., Lubensky T.C., Yodh A.G. (2014). Chiral symmetry breaking and surface faceting in chromonic liquid crystal droplets with giant elastic anisotropy. Proc. Natl. Acad. Sci. USA.

[9] Lee H., Labes M.M. (1982). Lyotropic Cholesteric and Nematic Phases of Disodium Cromoglycate in Magnetic Fields. Mol. Cryst. Liq. Cryst..

[10] Labes M.M., Lee H. (1984). Helical Twisting Power of Amino Acids in a Nematic Lyophase. Mol. Cryst. Liq. Cryst..

[11] Shirai T., Shuai M., Nakamura K., Yamaguchi A., Naka Y., Sasaki T., Clark N.A., Le K.V. (2018). Chiral lyotropic chromonic liquid crystals composed of disodium cromoglycate doped with water-soluble chiral additives. Soft Matter.

[12] Berride F., Troche-Pesqueira E., Weiss R.G., Navarro-Vázquez A., Cid M.M. (2021). Effect of the degree of protonation of amino acid dopants in Chirality amplification in DSCG Lyotropic Nematic phases. Org. Chem..

[13] Pellegrino C., De Santo M.P., Spina L., Ciuchi F. (2021). Induced Chiral Chromonics Confined in Micrometric Droplets. Adv. Funct. Mater..

[14] Bao P., Paterson D.A., Peyman S.A., Jones J.C., Sandoe J.A.T., Gleeson H.F., Evans S.D., Bushby R.J. (2021). Production of giant unilamellar vesicles and encapsulation of lyotropic nematic liquid crystals. Soft Matter.

[15] Tixier T., Heppenstall-Butler M., Terentjev E.M. (2005). Stability of cellulose lyotropic liquid crystal emulsions. Eur. Phys. J. E.

[16] Petriashvili G., De Santo M.P., Hernandez R.J., Barberi R., Cipparrone G. (2017). Mixed emulsion of liquid crystal microresonators: Towards white laser systems. Soft Matter.

[17] De Gennes P.G., Prost J. (1993). The Physics of Liquid Crystals.

[18] Nakayama K., Ohtsubo J. (2012). Optical security device providing fingerprint and designed patternindicator using fingerprint texture in liquid crystal. Opt. Eng..

[19] Lopez-Leon T., Fernandez-Nieves A. (2011). Drops and shells of liquid crystal. Colloid Polym. Sci..

[20] Lydon J. (2011). Chromonic liquid crystalline phases. Liq. Cryst..

[21] Tone C.M., De Santo M.P., Buonomenna M.G., Golemme G., Ciuchi F. (2012). Dynamical homeotropic and planar alignments of chromonic liquid crystals. Soft Matter.

[22] Urbanski M., Reyes C.G., Noh J., Sharma A., Geng Y., Jampani V.S.R., Lagerwall J.P.F. (2017). Liquid crystals in micron-scale droplets, shells and fibers. J. Physics: Condens. Matter.

[23] Thirumurugan R., Anitha K. (2017). Growth, structural, physical and computational perspectives of trans-4-hydroxy-l-proline: A promising organic nonlinear optical material with large laser-induced damage threshold. Mater. Res. Express.

